# Prevalence and risk factors of monochorionic diamniotic twinning after assisted reproduction: A six-year experience base on a large cohort of pregnancies

**DOI:** 10.1371/journal.pone.0186813

**Published:** 2017-11-06

**Authors:** Bing Song, Zhao-Lian Wei, Xiao-Feng Xu, Xue Wang, Xiao-Jin He, Huan Wu, Ping Zhou, Yun-Xia Cao

**Affiliations:** 1 Reproductive Medicine Center, Department of Obstetrics and Gynecology, The First Affiliated Hospital of Anhui Medical University, Hefei, China; 2 Province Key Laboratory of Reproductive Health and Genetics, AHMU, Hefei, China; 3 Anhui Provincial Engineering Technology Research Center for Biopreservation and Artificial Organs, Hefei, China; Peking University Third Hospital, CHINA

## Abstract

**Objective:**

To characterize the incidence and risk factors for monochorionic diamniotic(MC-DA) twinning after assisted reproductive technologies (ART).

**Design:**

Retrospective population-based study.

**Setting:**

The study was conducted in China; Department of Reproductive Medicine Center at The First Affiliated Hospital of Anhui Medical University.

**Population:**

A cohort of 8860 clinical pregnancies after embryo transfer (ET) carried out in our center between 2011 and 2016 were retrospectively analyzed for the incidence of MC-DA twinning.

**Methods:**

Logistic regression was used to model the effect on the incidence of MC-DA twinning after ART. Different clinical data (maternal age) and laboratory procedures (type of ET (fresh versus frozen), insemination (IVF or ICSI)), embryo stage at time of ET (cleavage or blastocyst)) on the incidence of MC-DA twinning were evaluated.

**Main outcome measures:**

Monochorionic-diamniotic pregnancies were identified when more than one fetal poles was visualized in one gestational sac via trans-vaginal ultrasound at early first-trimester (7 to 8 weeks).

**Results:**

The overall MC-DA twinning rate was 2.55% (226/8860). Eighty one MC-DA twinnings occurred in the fresh cycles and 145 in the frozen cycles (2.67% vs. 2.49%). MC-DA twinning incidence showed no significant difference whether ICSI was performed or not (2.79% vs. 2.44%). The MZT that resulted from single embryo transfer (SET) cycles (1.99%) was slightly lower than multiple embryo transfer cycles (2.61%),but with non-significance. However, women <35 years displayed a significant higher rate (2.81%) than women ≥35 years old (1.16%). Blastocyst transfer was associated with a significantly increase in MC-DA twinning incidence than cleavage-stage embryos transfer (2.79% VS 2.02%, *P* = 0.008). In the results of logistic regression analysis, blastocyst transfer is a major risk factor of MZT in the fresh cycles (*P* = 0.044), while maternal age plays a more important role in the frozen cycles (*P* = 0.004).

**Conclusions:**

There is an elevated prevalence of MC-DA twinning after ART. Both maternal age and blastocyst transfer are risk factors of monozygotic pregnancy independently. Blastocyst transfer is a major risk factor of MC-DA twinning in the fresh cycles, while maternal age plays a more important role in the frozen cycles.

## Introduction

More and more evidence show that monozygotic twinning (MZT) occur more often after assisted reproductive technologies (ART) than after spontaneous conceptions[1–5]. According to the embryo splitting time, there can be classified with three types of monozygotic: dichorionic-diamniotic(DC-DA), monochorionic-diamniotic (MC-DA) and monochorionic-monoamniotic (MC-MA). Of which, monochorionic-diamniotic (MC-DA) twin occupies most of the proportion of the incidence in monozygotic twinning[6–9]. Monochorionic twinning is generally associated with many maternal and fetal complications, including twin–twin transfusion syndrome, umbilical cord accidents, twin anemia–polycythemia syndrome, and fetal anomalies, compared with dichorionic pregnancies. Perinatal mortality rates are always higher with MC-DA twins than with dichorionic twins[6, 7, 10, 11].

Although the exact mechanisms involved in the increased incidence of MZT in the group of patients treated with ART is still not clear yet, there have be several publications address towards the potential reasons for this phenomenon. Previous studies have pointed out that the use of gonadotropin ovarian hyper stimulation [12], age of the patient [4, 13, 14], micro-manipulation techniques of zona pellucida, such as occurs with AH,PGD and ICSI[4, 15–17], blastocyst transfer[14, 18–21], culture medium conditions[22], genetics [9, 23], and embryo cohort quality [24]may confer the risk for developing MZT pregnancies. However, no general agreement on the cause has been reached to date [1, 2, 14, 24, 25].

Therefore, we performed a retrospective study of the embryological and clinical data in a large cohort of clinical pregnancies after ART in our center, in order to examine the potential risk factors between the ART procedures and specific patient characteristics.

## Materials and methods

### Study population

This study retrospectively reviewed all clinical pregnancies from fresh or frozen embryo transfer (ET) cycles with autologous oocytes performed in our center between January 2011 and August 2016. In order to standardize the embryo cohort quality analysis, donor oocyte cycles were excluded. All patients underwent ovulation induction, ultrasound-guided oocyte retrieval, conventional IVF or ICSI, and embryos transfer. Transfer order was determined by patients and their physician, in consultation with the embryology team. This study was approved by the institutional review board of the First Affiliated Hospital, Anhui Medical University.

### Clinical outcome

All pregnant patients were confirmed by detection of fetal heart activity on the use of trans-vaginal ultrasound by early first-trimester (7 to 8 weeks). Monozygotic twinning pregnancies were identified when the number of fetal heart beats exceeded the number of embryos transferred, and MC-DA pregnancies were identified when more than one fetal poles was visualized in one gestational sac (GS). Mono-amniotic twin pregnancies were characterized by the presence of a single amniotic cavity and yolk sac within one GS with one chorionic, this type of monozygotic twinning pregnancies were very rare[8].Ultrasound detection of monozygotic and dizygotic twins may be in error when more than one embryo was transferred. As they share the same obstetrical outcome, we laid the focus on the MC-DA pregnancies.

### Data analysis

Maternal age, micro-manipulation techniques (ICSI), length of culture (cleavage or blastocyst-stage), and the number of embryos transferred were analyzed between the MZ and non-MZ groups. MZT rates per ET were evaluated using multivariable logistic regression to account for the above-mentioned study parameters. We also performed a subgroup analysis dividing the sample according to the type of ET. In the subgroup of frozen embryo transfer(F-ET) cycles, whether hormone replacement associated with MZT rates or not was also analyzed. In all cases, a *P*-value was considered significant if <0.05. For the statistical analysis, we used Stata Software version 13.1 (Stata Corp, College Station, Texas,USA).

## Results

### The incidence of MZ twinning

**Fig 1:**
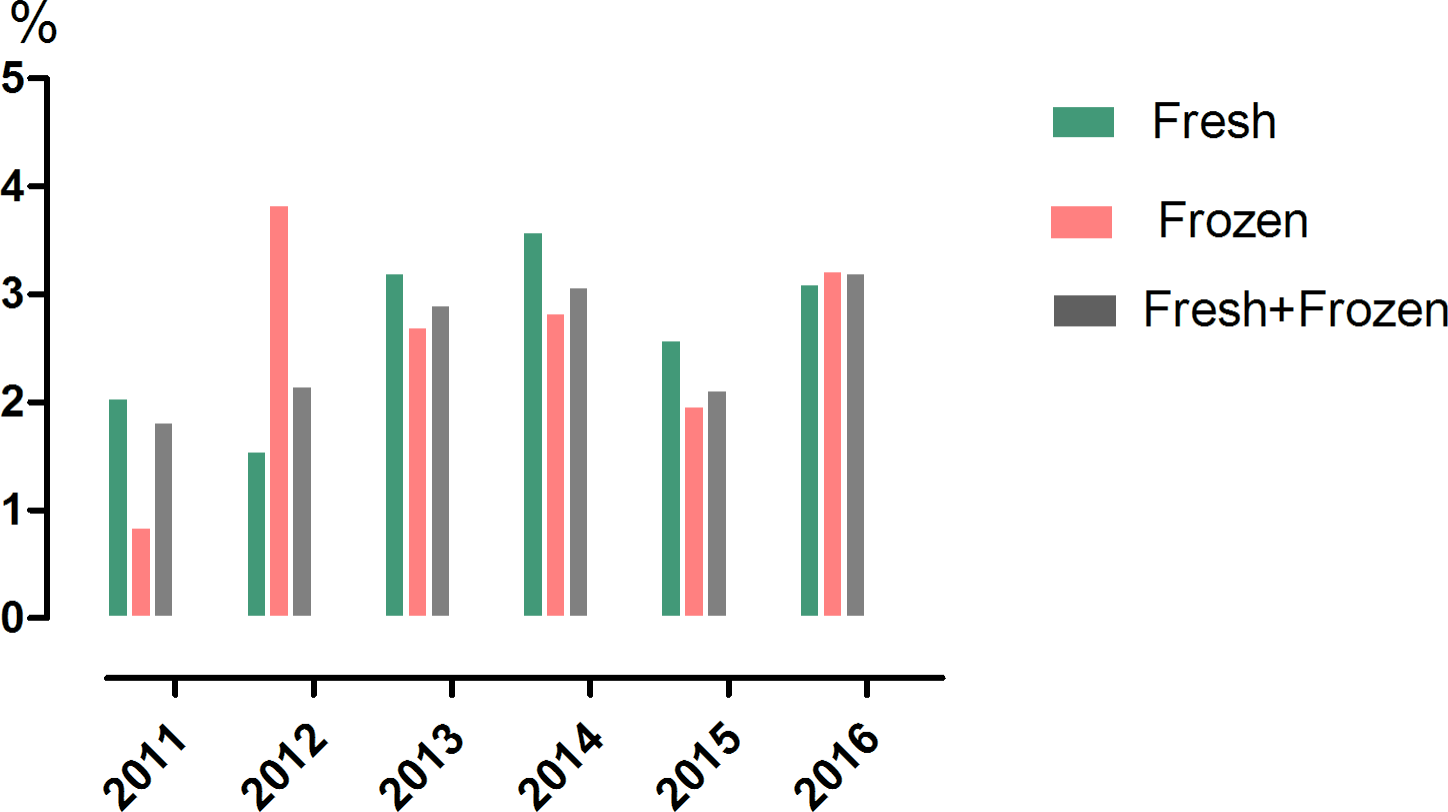
The incidence of monochorionic diamniotic(MC-DA) twinning in the last six years. Trends in MC-DA twinning for fresh, frozen and all embryo transfer cycles from 2011 to 2016.

From January 2011 to August 2016, 8860 pregnancies (3034 resulting from fresh embryos transfer cycles and 5826 from frozen cycles) were conceived. The overall incidence of MZ twinning among all clinical pregnancies was 2.55% (226/8860). Fig 1 showed the incidence of MZT from 2011 to 2016 in our center, a very high MZT rate range from 1.82% to 3.2% of clinical pregnancies were found in the latest 6 years.(Fig 1). When categorized according to the type of cycle attempted, the incidence was 2.67% (81/3034) for fresh embryo transfer cycles, and 2.49% (145/5826) for frozen–thawed embryo transfer cycles(Table 1). The couples who underwent ICSI had a similar MZT rate(2.44%, 149/6099) with couples who underwent conventional IVF (2.79%,77/2761). The MZT rate that resulted from single embryo transfer (SET) cycles (1.99%) was slightly lower than multiple embryo transfer cycles (2.61%), but with non-significance. Of note is that the rate of MZT with blastocyst transfer (2.79%) was significantly higher than cleavage-stage embryos transfer (2.02%, *P* = 0.008). When compared with older maternal age(≥35 years), MZT is more likely to happen in embryo transfer to females with younger age(*P* = 0.001).

**Table 1:** Analysis of monozygotic twin (MZT) risk factors by multivariate logistic regression.

	MZT(MC-DA) (N = 226)%	Non-MZT (N = 8634)%	total	*P*	OR[95%CI]
Type of cycles				0.134	0.798[0.594–1.072]
Fresh cycles	81(2.67%)	2953(97.33%)	3034		
Frozen- cycles	145(2.49%)	5681(97.51%)	5826		
Maternal Age				**0.001**	**0.412[0.247–0.687]**
<35 years old	210(2.81%)	7268(97.19%)	7478		
≥35 years old	16(1.16%)	1368(98.84%)	1384		
Insemination				0.302	1.159[0.876–1.533]
Conventional IVF	149(2.44%)	5951(97.56%)	6100		
ICSI	77(2.79%)	2683(97.21%)	2760		
SET or not				0.186	1.419[0.845–2.384]
SET	16(1.99%)	788(98.01%)	804		
Multi-ET	210(2.61%)	7846(97.39%)	8056		
Embryo stage				**0.008**	**1.568[1.127–2.181]**
Cleavage	56(2.02%)	2719(97.98%)	2775		
Blastocyst	170(2.79%)	5915(97.21%)	6085		

MC-DA, monochorionic-diamniotic; SET, Single embryo transfer; OR, odds ratio; CI, confidence interval; Boldface: *P*<0.05

### Risk factors in the subgroups included fresh and frozen cycles

In an attempt to identify potential risks of MZT pregnancy, we performed a series of studies to examine the relationships between MZT pregnancy and various ART procedures. Since the circumstances of these patients differed, we divided the data into two groups according to the type of embryo transfer (ET). In the fresh ET group, we found that monozygotic twinning occurred in 30 of 1001 cycles (3.00%) with ICSI and 51 of 2033(2.51%) with conventional IVF (Table 2). We failed to find significant difference between the two groups. When stratified by SET or not, it seemed that SET cycles had a trend to a lower risk of MZT but with no statistical difference (1.72%vs 2.75%).The incidence of MZT pregnancy was higher in cycles with maternal age younger than 35, but with non-significant difference (2.91% vs.1.62%; *P* = 0.071).The transfer of a blastocyst was associated with an increased possibility of MZT pregnancy compared to cleavage-stage ET (3.26% vs 2.19%; *P* = 0.044). The same analysis were repeated for frozen–thawed embryo cycles (Table 3). We could not find any links between the incidence of MZT pregnancy and ICSI, SET and hormone replacement cycles. It can be inferred blastocyst transfer in FET cycles have higher incidence of MZT, but with no statistical difference (2.66% vs 1.74%, *P* = 0.069). The incidence of MZT pregnancy was significantly higher in cycles performed in women <35 years of age (2.75% vs. 1.01%; *P* = 0.004).

**Table 2:** Risk factors of monozygotic twin (MZT) in the fresh cycles.

	MZT(MC-DA) (N = 81)%	Non-MZT (N = 3034)%	total	*P*	OR[95%CI]
Maternal Age				0.071	0.487[0.223–1.063]
<35 years old	74(2.91%)	2466(97.09%)	2540		
≥35 years old	8(1.62%)	486(98.38%)	494		
Insemination				0.358	1.240[0.783–1.963]
Conventional IVF	51(2.51%)	1982(97.49%)	2033		
ICSI	30(3.00%)	971(97.00%)	1001		
SET or not				0.241	1.846[0.663–5.141]
SET	4(1.72%)	229(98.28%)	233		
Multi-ET	77(2.75%)	2724(97.25%)	2801		
Embryo stage				**0.044**	**1.586[1.013–2.484]**
Cleavage	37(2.19%)	1649(97.81%)	1686		
Blastocys	44(3.26%)	1304(96.74%)	1348		

MC-DA, monochorionic-diamniotic; SET, Single embryo transfer; OR, odds ratio; CI, confidence interval; Boldface: *P*<0.05

**Table 3:** Subgroup analysis of different ART parameters on MZT incidence in the frozen-thawed cycles.

	MZT(MC-DA) (N = 145)%	Non-MZT (N = 2953)%	total	*P*	OR[95%CI]
Maternal Age				**0.004**	**0.366[0.186–0.722]**
<35 years old	136(2.75%)	4802(97.25%)	4938		
≥35 years old	9(1.01%)	879(98.99%)	888		
				0.871	1.028[0.735–1.437]
Natural cycle	61(2.50%)	2377(97.50%)	2438		
Hormone replacement	84(2.48%)	3304(97.52%)	3388		
Insemination				0.585	1.014[0.774–1.574]
Conventional IVF	98(2.41%)	3969(97.59%)	4067		
ICSI	47(2.67%)	1712(97.33%)	1759		
SET or not				0.446	1.265[0.691–2.314]
SET	12(2.10%)	559(97.90%)	571		
Multi-ET	133(2.53%)	5122(97.47%)	5255		
Embryo stage				0.069	1.576[0.965–2.574]
Cleavage	19(1.74%)	1070(98.26%)	1089		
Blastocyst	126(2.66%)	4611(97.34%)	4737		

MC-DA, monochorionic-diamniotic; SET, Single embryo transfer; OR, odds ratio; CI, confidence interval; Boldface: *P*<0.05

## Discussion

MZT pregnancies are associated with an increased risk of maternal and fetal complications [7]. It is proved that monochorionic twins are associated with a higher perinatal morbidity and perinatal mortality rate than dizygotic twin pregnancies[10]. In our large cohort study, we reported the overall incidence of MC-DA monozygotic twins approached 2.55% of all clinical pregnancies after all embryo transfers cycles, including fresh and frozen–thawed embryo transfers. The data shows at least a five to six-fold rise in the incidence of MC-DA monozygotic twins after assisted reproductive techniques (ART) in the Chinese population, compared with natural conception[5,8].

According to previous study, the thickness of the zona pellucida (ZP) was attenuation with increased age in women, which could make the embryo more vulnerable to ICM splitting[2,14]. Later maternal age was considered as a risk factor of increasing the incidence of monozygotic twinning in natural cycles [2, 14]. However, we found that women <35 years old were more likely to experience MZT than women older than 35 years, comparing to many other studies [13, 14]. Actually, we observed a nearly threefold increase in MZT after F-ET cycles (2.75% vs. 1.01%). This result may suggest the exact mechanism of high MZT rates after ART was different from natural conception. We inferred younger maternal age may have good-quality embryos which could be more likely to cause ICM splitting. Younger maternal age may be an independent risking factor of the incidence of MZT after ART, especially in FET cycles.

It has prompted discussion of a possible connection between micro-manipulation techniques (such as ICSI, AH, PGD) and increased MZT [15, 17, 26–28]. All the techniques manipulate the ZP in different ways and leave small defects in the ZP, which may cause ICM splitting and MZ twinning. In our center, assisted hatching is a routine application in the frozen cycles since 2011. Our previous and this study showed that this technique has no effect on MZT[29]. The zona of frozen embryos seems to be harder than fresh embryos. Our data failed to show any distinguishing characteristic of MZ twin conceptions with respect to frozen-thawed procedure and simple microscopic insemination technology (ICSI). Our study suggested that zona penetration (especially ICSI) and cryopreservation should not be included as the MZT risk factors. It seemed that embryos had a quick repair system when zona was slightly injured.

It has been widely reported that prolonged culture embryos to the blastocyst stage may facilitate embryo selection, reduce aneuploidy embryos and improve live birth rates. More and more studies reported an association between the incidence of MZT and blastocyst transfer [18,20,26, 30]. Accordingly, we also reported a 2.79% of pregnancies generated by blastocyst transfer were complicated by MZT, which was significantly higher than embryos transferred from cleavage stage (2.02%, *P* = 0.008). Although blastocyst transfer in FET cycles have a higher incidence of MZT than cleavage stage, but shows no statistical difference (2.66% vs 1.74%, *P* = 0.069). This divergence may be attributed to the limited cases of MZT. Though many theories explaining about the possible mechanism about how blastocyst transfer affects the MZT rate were focused on hardening of the ZP and culture media composition, there are more studies attributed this phenomenon on the high-quality embryos. They suggested that prolonged culture embryos may be more sensitive to the effects of mechanical manipulation or to changes in temperature and pH during monitoring, which might result in higher rates of MZT after blastocyst transfer. Recently, the data from a large cohort study showed that embryo cohort quality may be a key factor that explains the elevated incidence of MZT in blastocyst transfer [24]. In their opinion, blastocyst transfer is not associated with increased rates of monozygotic twins when controlling for embryo cohort quality.

In conclusion, the present study demonstrates a higher rate of monochorionic diamniotic(MC-DA) twinning after ART compared with spontaneous pregnancies. Patients <35 years old and blastocyst transfer appears to be significantly associated with increased rates of MZT. Younger maternal age may be the main factor of MZT in the frozen cycles while blastocyst transfer played a more important role of the incidence of MZT in the fresh cycles. In view of the complications arising from MZT pregnancies, the high rate of MZT and the related risking factors should be taken into account before ART treatment.

## References

[1] Aston KI, Peterson CM, Carrell DT. Monozygotic twinning associated with assisted reproductive technologies: a review. Reproduction. 2008;136(4):377–86. doi: 10.1530/REP-08-0206 . 18577552 10.1530/REP-08-0206

[2] Sobek A, Prochazka M, Klaskova E, Lubusky M, Pilka R. High incidence of monozygotic twinning in infertility treatment. Biomed Pap Med Fac Univ Palacky Olomouc Czech Repub, 2016 160(3): 358–62. doi: 10.5507/bp.2016.016 27049533 10.5507/bp.2016.016

[3] Blickstein I, Verhoeven HC, Keith LG. Zygotic splitting after assisted reproduction. The New England journal of medicine. 1999;340(9):738–9. doi: 10.1056/NEJM199903043400916 . 10068338 10.1056/NEJM199903043400916

[4] Alikani M, Cekleniak NA, Walters E, Cohen J. Monozygotic twinning following assisted conception: an analysis of 81 consecutive cases. Human reproduction. 2003;18(9):1937–43. . 12923153 10.1093/humrep/deg369

[5] Mateizel I, Santos-Ribeiro S, Done E, Van Landuyt L, Van de Velde H, Tournaye H, et al Do ARTs affect the incidence of monozygotic twinning? Human reproduction. 2016 doi: 10.1093/humrep/dew216 . 27664211 10.1093/humrep/dew216

[6] Derom R, Bryan E, Derom C. Keith L, Vlietinck R. Twins, chorionicity and zygosity. Twin Res, 2001 4(3): 134–6. 11665310 10.1375/1369052012344

[7] Chauhan SP, Scardo J A, Haye E, Abuhama AZ, Berghella V. Twins: prevalence, problems, and preterm births. Am J Obstet Gynecol, 2010 203(4):305–15. doi: 10.1016/j.ajog.2010.04.031 20728073 10.1016/j.ajog.2010.04.031

[8] Hall JG. Twinning. Lancet. 2003;362(9385):735–43. doi: 10.1016/S0140-6736(03)14237-7 . 12957099 10.1016/S0140-6736(03)14237-7

[9] Steinman G. Mechanisms of twinning. VI. Genetics and the etiology of monozygotic twinning in in vitro fertilization. The Journal of reproductive medicine. 2003;48(8):583–90. . 12971137

[10] Dube J, Dodds L, Armson BA. Does chorionicity or zygosity predict adverse perinatal outcomes in twins? American journal of obstetrics and gynecology. 2002;186(3):579–83. . 11904627 10.1067/mob.2002.121721

[11] Benirschke K. The monozygotic twinning process, the twin-twin transfusion syndrome and acardiac twins. Placenta. 2009;30(11):923–8. doi: 10.1016/j.placenta.2009.08.009 . 19748667 10.1016/j.placenta.2009.08.009

[12] Derom C, Vlietinck R, Derom R, Van den Berghe H, Thiery M. Increased monozygotic twinning rate after ovulation induction. Lancet. 1987;1(8544):1236–8. . 2884372 10.1016/s0140-6736(87)92688-2

[13] Abusheikha N, Salha O, Sharma V, Brinsden P. Monozygotic twinning and IVF/ICSI treatment: a report of 11 cases and review of literature. Human reproduction update. 2000;6(4):396–403. . 10972526 10.1093/humupd/6.4.396

[14] Knopman J, Krey LC, Lee J, Fino ME, Novetsky AP, Noyes N. Monozygotic twinning: an eight-year experience at a large IVF center. Fertil Steril, 2010 94(2): 502–10. doi: 10.1016/j.fertnstert.2009.03.064 19409556 10.1016/j.fertnstert.2009.03.064

[15] Sills ES, Moomjy M, Zaninovic N, Veeck LL, McGee M, Palermo GD, et al Human zona pellucida micromanipulation and monozygotic twinning frequency after IVF. Human reproduction. 2000;15(4):890–5. . 10739838 10.1093/humrep/15.4.890

[16] Skiadas CC, Missmer SA, Benson CB, Gee RE, Racowsky C. Risk factors associated with pregnancies containing a monochorionic pair following assisted reproductive technologies. Human reproduction. 2008;23(6):1366–71. doi: 10.1093/humrep/den045 . 18378561 10.1093/humrep/den045

[17] Verpoest W, Van Landuyt L, Desmyttere S, Cremers A, Devroey P, Liebaers I. The incidence of monozygotic twinning following PGD is not increased. Hum Reprod, 2009 24(11): 2945–50. doi: 10.1093/humrep/dep280 19661123 10.1093/humrep/dep280

[18] Kawachiya S, Bodri D, Shimada N, Kato K, Takehara Y, Kato O. Blastocyst culture is associated with an elevated incidence of monozygotic twinning after single embryo transfer. Fertil Steril, 2011 95(6): 2140–2. doi: 10.1016/j.fertnstert.2010.12.018 21215395 10.1016/j.fertnstert.2010.12.018

[19] Wright V, Schieve LA, Vahratian A, Reynolds MA. Monozygotic twinning associated with day 5 embryo transfer in pregnancies conceived after IVF. Human reproduction. 2004;19(8):1831–6. doi: 10.1093/humrep/deh338 . 15192064 10.1093/humrep/deh338

[20] Chang HJ, Lee JR, Jee BC, Suh CS, Kim SH. Impact of blastocyst transfer on offspring sex ratio and the monozygotic twinning rate: a systematic review and meta-analysis. Fertility and sterility. 2009;91(6):2381–90. doi: 10.1016/j.fertnstert.2008.03.066 . 18718582 10.1016/j.fertnstert.2008.03.066

[21] Milki AA, Jun SH, Hinckley MD, Behr B, Giudice LC, Westphal LM. Incidence of monozygotic twinning with blastocyst transfer compared to cleavage-stage transfer. Fertility and sterility. 2003;79(3):503–6. . 12620430 10.1016/s0015-0282(02)04754-4

[22] Sparks AE. Culture systems: embryo culture and monozygotic twinning. Methods in molecular biology. 2012;912:387–97. doi: 10.1007/978-1-61779-971-6_22 . 22829386 10.1007/978-1-61779-971-6_22

[23] Sobek A, Zborilova B, Prochazka M, Silhanova E, Koutna O, Klaskova E, et al High incidence of monozygotic twinning after assisted reproduction is related to genetic information, but not to assisted reproduction technology itself. Fertil Steril, 2015 103(3): 756–60. doi: 10.1016/j.fertnstert.2014.12.098 25583445 10.1016/j.fertnstert.2014.12.098

[24] Franasiak JM, Dondik Y, Molinaro TA, Hong KH, Forman EJ, Werner MD, et al Blastocyst transfer is not associated with increased rates of monozygotic twins when controlling for embryo cohort quality. Fertil Steril, 2015 103(1): 95–100. doi: 10.1016/j.fertnstert.2014.10.013 25455537 10.1016/j.fertnstert.2014.10.013

[25] Papanikolaou EG, Fatemi H, Venetis C, Donoso P, Kolibianakis E, Tournaye H, et al Monozygotic twinning is not increased after single blastocyst transfer compared with single cleavage-stage embryo transfer. Fertil Steril, 2010 93(2): 592–7. doi: 10.1016/j.fertnstert.2008.12.088 19243755 10.1016/j.fertnstert.2008.12.088

[26] Tarlatzis BC, Qublan HS, Sanopoulou T. Zepiridis L. Grimbizis G, Bontis J. Increase in the monozygotic twinning rate after intracytoplasmic sperm injection and blastocyst stage embryo transfer. Fertil Steril, 2002 77(1): 196–8. 11779618 10.1016/s0015-0282(01)02958-2

[27] Behr B, Milki AA. Visualization of atypical hatching of a human blastocyst in vitro forming two identical embryos. Fertility and sterility. 2003;80(6):1502–3. . 14667890 10.1016/j.fertnstert.2003.07.001

[28] Allegra A, Sammartano F, Scaglione P, Coffaro F, Marino A, Volpes A.Monozygotic bichorionic twinning after transfer of a single frozen/thawed embryo that has undergone quarter laser-assisted zona thinning: a case report. J Assist Reprod Genet, 2005 22(11–12): 437–41. doi: 10.1007/s10815-005-7483-9 16331542 10.1007/s10815-005-7483-9 PMC3455153

[29] Tingting L, Dongmei J, Dawei C, Yunxia C. Effects of laser-assisted hatching on the outcomes of the frozen-thawed blastocyst transfer. Acta Universitatis Medicinalis Anhui. 2015;50(6):749–52.

[30] Behr B, Fisch JD, Racowsky C, Miller K, Pool TB, Milki AA. Blastocyst-ET and monozygotic twinning. Journal of assisted reproduction and genetics. 2000;17(6):349–51. doi: 10.1023/A:1009461213139 ; PubMed Central PMCID: PMC3455394. 11042833 10.1023/A:1009461213139 PMC3455394

